# Mathematical optimization models for reallocating and sharing health equipment in pandemic situations

**DOI:** 10.1007/s11750-022-00643-3

**Published:** 2022-09-02

**Authors:** Víctor Blanco, Ricardo Gázquez, Marina Leal

**Affiliations:** 1 Institute of Mathematics (IMAG), Universidad de Granada, Granada, Spain; 2 Dpt. Quant. Methods for Economics & Business, Universidad de Granada, Granada, Spain; 3 Dpt. Statistics, Mathematics and Informatics, Universidad Miguel Hernández, Elche, Spain; 4 Centro de Investigación Operativa (CIO), Universidad Miguel Hernández, Elche, Spain

**Keywords:** Reallocation, Sharing policies, Robust integer linear programming, Math-Heuristic, COVID-19, 91B32, 90C15, 90C10, 90B15

## Abstract

In this paper we provide a mathematical programming based decision tool to optimally reallocate and share equipment between different units to efficiently equip hospitals in pandemic emergency situations under lack of resources. The approach is motivated by the COVID-19 pandemic in which many Heath National Systems were not able to satisfy the demand of ventilators, sanitary individual protection equipment or different human resources. Our tool is based in two main principles: (1) Part of the stock of equipment at a unit that is not needed (in near future) could be shared to other units; and (2) extra stock to be shared among the units in a region can be efficiently distributed taking into account the demand of the units. The decisions are taken with the aim of minimizing certain measures of the non-covered demand in a region where units are structured in a given network. The mathematical programming models that we provide are stochastic and multiperiod with different robust objective functions. Since the proposed models are computationally hard to solve, we provide a *divide-et-conquer* math-heuristic approach. We report the results of applying our approach to the COVID-19 case in different regions of Spain, highlighting some interesting conclusions of our analysis, such as the great increase of treated patients if the proposed redistribution tool is applied.

## Introduction

We recently lived a pandemic situation caused by the COVID-19 virus. The high contagiousness of this virus provoked its rapid propagation all over the world; and this led to the sudden need of hospitalization of a large amount of patients affected by COVID-19, many of them in Intensive Care Units (ICU). This abrupt and massive increase in the number of hospitalizations brought some hospitals to the collapse and produced lack of material and human resources, such as diagnostic tests, ventilators, ICU beds, personal protective equipment for sanitarians, etc., in many of them. This extreme situation gave rise to the application of improvised measures, such as the creation of field hospitals, the use of non-approved personal protective equipment or the application of ageisming when assigning (insufficient) ventilators to patients (Cesari and Proietti 2020; García-Soler et al. 2020), even though this last measure is not allowed by the World Health Organization (World Health Organization n.d.). Other improvised measure, in Spain for instance, were the hire of ventilators between hospitals in different cities just to cover the demand at a specific time of the pandemic, without taking into account demand forecasting (Libertad Digital 2020; Hoy 2020; El Médico Interactivo 2020; El País 2020), or distributing test packages between regions by population instead of by the number of infected citizens (Global 2020). In these pandemic situations, like the one caused by the COVID-19, in which the number of infected citizens and the gravity of their disease are distributed heterogeneously in a country or state, it is a must to provide efficient strategies to distribute equipment and share the available resources between different hospitals or units in order to efficiently equip these hospitals or field hospitals to satisfy the demand of the patients and sanitarians.

Furthermore, during the COVID-19 pandemic, patients were also moved from collapsed hospitals to hospitals with a lower level of saturation. However, as the Spanish Society of Urgency and Emergency Medicine reported, moving patients between hospitals affects patients’ safety and even influence patient deterioration and mortality (Sociedad Española de Medicina de Urgencias y Emergencias 2020). This fact emphasizes the need of distributing and sharing health equipment to properly equip hospitals to avoid patients translation in these collapsed situations. The need of (re)distribution of health equipment is also strengthen by the fact that most of the ICU are expanded in most of the hospitals in pandemic situation, as we learned in the recent COVID-19 pandemic, which led to the requirement of additional equipment.

### Discussion

Allocating/reallocating resources is a recurrent application of Operations Research and a wide variety of situations and tools analyzing the efficient distribution of goods can be found in literature (see, e.g Baricelli et al. 1996; Bodson 2002; Gomar et al. 2002; Hegazy 1999; Michaud and Michaud 2008; Wang et al. 2011, among many others). In the case of sanitary emergency situations, many papers have been recently published proposing methods to deal with the logistics and transportation of goods during the COVID-19 pandemic (see Chowdhury et al. (2021) for a recent review on the topic). In particular, there are some of them proposing different approaches to allocate scarce resources. Arora et al. (2010) provide a nonlinear optimization model to allocate antivirals among different regions of the United States in order to share resources among regions using a central stockpile minimizing a measure of the lost benefits due to resource shortages. In a recent paper, Mehrotra et al. (2020) propose a stochastic multiperiod planning model to allocate and reallocate ventilators to treat critical patients. That model is designed for a particular network and assumes that a central agency makes decisions on the reallocation of ventilators while federal agencies and states decide on the percentage of available ventilators to share, by minimizing the expected non-covered demand. Yin et al. (2020) provide linear programming-based methodologies for multi-resource allocation to hospitals, where resources are grouped in different classes, each of them with particular characteristics. A biobjective optimization problem considering the minimization of both ventilator-day shortages and inter-unit transfers is proposed by Bertsimas et al. (2021) to give response to COVID-19. In Rastegar et al. (2021), the authors propose a mixed integer linear model for the equitable flu vaccine distribution to high-risk people in developing countries. Another example of the recent developments on optimal resource allocation in pandemic situations is Dönmez et al. (2022) where the number of infections (as a function of the number of workers in a hospital not using personal protective equipment–PPE) and the deprivation cost is minimized when allocating PPE to hospitals.

Besides, many of the research on resource allocation is cost-oriented, i.e., the decision aid tools are designed to minimize, among others, certain function of the transportation or set-up costs. This is counterproductive in emergency situations, where satisfying in adequate time the needs of the society affected by any damage, beyond its distribution cost, is crucial. Thus, an efficient resource allocation and collaboration among the different units which may supply materials is essential. In the COVID-19 situation, the life of sick patients depends on the use of ventilators, and the non-infection of health personnel depends on the availability of sanitary protection equipment. Therefore, designing strategies to share equipment between hospitals will help to reduce the impact of this crisis and avoid a lack of human resources being able to cut the pandemic. Furthermore, national and regional governments were receiving extra stock of equipment, provided by international suppliers or by different private initiatives (as the home-made confection of face-masks, 3D-printing of helmets, etc.) and sharing this stock among the different units should be done by means of the demand of each of the units instead of population-based sharing, as those that have been applied. The COVID-19 pandemic has already provoked around to six million worldwide deaths until mid-2022, which evidences the need of cooperation of the different agents to palliate the effect of the virus.

#### Main contributions

The main goal of this paper is to develop a general framework for a rapid and efficient reallocation and sharing of any type of necessary health equipment, unlike described works focused on particular goods, as for instance the one by Arora et al. (2010). This reallocation is made between different sanitary units in a pandemic or in other situations in which the resources are scarce and should be urgently distributed. Our approach assumes general networks structures for the distribution, generalizing in this way some of the approaches that have been recently proposed , including them as special cases, in particular, the one of Mehrotra et al. (2020). Unlike classical resource (re)allocation literature, we minimize different functions of the non-covered demand in a framework which considers four main ingredients:

- *Distribution Network*: The units and the different types of available logistic platforms are linked in a network-based input information, allowing us to model different policies established for the regions in a country. We hence assume that products can be distributed through given general network structures.
- *Multiperiod*: The planning is performed for a short time horizon, being the approach a multiperiod decision-making tool.
- *Uncertainty*: Since the demand of equipment in each unit is stochastic along time (it depends on the number of sick or infected citizen), we incorporate this uncertainty in the model.
- *Robust Decisions*: Our model decides on the optimal way to reallocate and share extra stock based on different aggregation measures of the non-covered demand along the planing horizon, minimizing different robust objective functions, as the maximum (by units) non-covered expected demand; the maximum (by time periods) non-covered expected demand, the maximum (by regions) non-covered expected demand; the overall non-covered expected demand^1^, and also their minimax regret counterparts that provide robust solutions with a *good* performance under any of the possible uncertain situations that may happen.

Apart from the above, our model has different particularities that can be adequately tuned to be adapted to each situation. We limit the number of deliveries from each unit to avoid the use of excessive resources to load, unload and mount the equipment. We also set an upper bound for the amount of equipment to be delivered from each unit to a percentage of the available stock at each period, allowing hospitals to fix such a percentage based on their risk level.

It is clear that Mathematical Programming plays an important role in the design of optimal strategies to (re)distribute goods and determine sharing policies. The above characteristics of our model would require to incorporate to our methodology different elements which makes the problem specially difficult to solve. On the one hand, our problem will be modeled as a stochastic programming problem (Birge and Louveaux 2011), in which a function of the expected non-covered demand (which is the uncertain data in our problem) is minimized. Second, in order to find fair solutions that do not harm the weakest units, we use different robust objective functions in our models of type minmax (Campbell et al. 2008; Ogryczak 2000; Puerto et al. 2018; Shahnejat-Bushehri et al. 2021; Sun et al. 2021; Ye et al. 2017) and minmax regret (Conde 2007; Conde et al. 2018; Gutiérrez et al. 1996; López-de-los Mozos et al. 2013). Also, our problem consists of a planning model in multiple periods, being necessary to deal with multiperiod problems (Albareda-Sambola et al. 2010; Ben Mohamed et al. 2020; Gholami et al. 2020; Pun and Wong 2019; Shin et al. 2019).

#### Organization

The rest of the paper is organized as follows. In Sect. 2, we describe the input elements to derive a model for the problem. Section 3 is devoted to present the mathematical programming models for the problem. The math-heuristic approach is described in Sect. 4. The analysis of our model for Spanish COVID-19 data is reported in Sect. 5. Finally, we draw some conclusions and further extensions in Sect. 6.

## Preliminaries

We analyze here the problem of distributing goods on a network with several particularities, as described above. In this section we describe the elements involved in the problems and introduce the notation and the problem under study.

Let us consider a weighted directed graph $G=(N,A; \mathbf {w})$ where:

- $N=\{1, \ldots , n\}$ represents the different units in the distribution system. Some nodes may represent hospitals or (local, regional or national) health logistic centers.
- *A* is the set of arcs, it indicates the available direct links between units.
- $\mathbf {w}$ is the set of weights, origin-destination times (in days) to transfer equipment between the arcs. These weights include the times needed to load, deliver, unload and mount the equipment.

For the sake of presentation we assume that a single type of product is distributed along the network, although our approach can be easily adapted to distribute different types of goods (in such a case, a set of weights for each product being distributed must be provided).

**Fig. 1:**
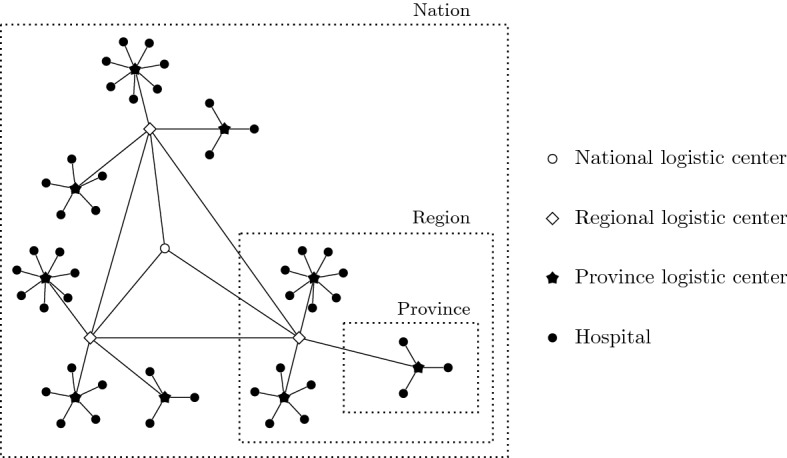
Example of the graph structure involving different types of units and links in a distribution system

In Fig. 1, we illustrate an example of a hierarchical network that may represent the situation in many countries when distributing health equipment. There, solid circles represent different hospitals. They are supplied by local logistic centers (stars) which are at the same time supplied by regional logistic centers (squares). All the regions are supplied by a national logistic center (empty circle). The lines indicate the links in which the products can be distributed. This network example shows the adaptability of the general network structure that we assume to particular network situations.

From the graph *G*, we consider the set of pairs of nodes for which there is a directed path in the graph between them, i.e.

$$\begin{aligned} W=\{(i,j): i,j\in N \text { such that there exist a path from }i\text { to }j\}. \end{aligned}$$

In order to provide an optimal sharing of extra stocks and to account for the different policies of the different regions, we assume that the set of nodes, *N*, is divided into *p* groups, $N_1, \ldots , N_p \subseteq N$, not necessarily disjoint, which may represent different regions or clusters of hospitals which may receive extra stock from the same source. Observe that one unit may belong to different regions and receive equipment from different sources. This would be the case of countries that centrally share equipment directly to all (or some) hospitals but also regions with extra or available stock share equipment to the hospitals in the region. In this case, a hospital in the region belongs to two of the *N*-sets, to the one in which all the hospitals belong to and also to the one in which only the hospitals of the regions are included.

In the example drawn in Fig. 1, the different circles linked to a same star may represent one of these sets (provinces), all which are connected to a star linked to the same square represent others sets (regions), and all the square nodes linked with an empty circle node is the last set (country). We denote by $P=\{1, \ldots , p\}$, the index set for the groups.

We also consider the following list of input parameters:

- $T = \{1, \ldots , q\}$: planning horizon (in days). We will perform a distribution planning for a finite number, *q*, of periods.
- $s_i^0$: initial stock at node *i*, for all $i\in N$. Each unit is assumed to have this nonnegative initial stock at the beginning of the planning horizon and that is available to be used or delivered. It represents the number of equipment at each unit to cover the demand.
- $q_k^t$: amount of extra stock to be shared in period *t* between the nodes in group $N_k$, for $k \in P$. We assume that the units on group $N_k$ may receive a nonnegative number of units at each period. It allows to model situations in which certain regions buy or receive equipment at certain periods and they want to share them among the units that belong to the region.
- $Q_i$: upper bound on the number of deliveries from node *i* to different units, for $i\in N$. In order to avoid a large (and unrealistic) number of deliveries from units with low demand but high initial stocks, we will restrict the deliveries from each unit. This upper bound will be induced by the capacity of each unit to prepare and load the equipment to deliver.
- $\ell _{ij}$: length of the shortest path in the graph with origin *i* and destination *j*, for all $(i,j)\in W$. This length represents the time (in days) needed to deliver an equipment from unit *i* to unit *j* on the graph *G*.
- $\gamma _i$: proportion of the available stock (after covering its demand) that unit *i* is willing to delivered to other units, for $i\in N$.
- $g_i$: upper bound on the number of equipment to be deliver from unit *i*, for $i\in N$.
- $a_i$: storage capacity of unit *i*, for $i\in N$.

Note that the parameters $Q_i$, $\gamma _i$, $g_i$ and $a_i$ do not depend on the time period. However, the time dependence could be easily incorporated in the model, if needed.

### Demands

Apart from the above deterministic information, we consider that the demands of equipment required by the units at each period are, as usual, uncertain. We denote the demands as $d(\xi )$, with $\xi$ a random variable. We assume that $\xi$ has finite support, i.e., that a finite number of possible realizations for the demands is possible. We consider the following notation for the demands,

- $\Omega (T)$: finite support of possible scenarios, $\omega \in \Omega (T)$. Abusing of notation, we will obviate the index set *T* in the definition of the set of scenarios, i.e., we will denote by $\Omega \equiv \Omega (T)$.
- $d_i^{t\omega }$: demand for node $i\in N$ at period $t\in T$ under scenario $\omega \in \Omega (T)$.
- $p^\omega$: probability of scenario $\omega \in \Omega (T)$, with $p^{\omega }\ge 0$, and $\sum _{\omega \in \Omega (T)} p^{ \omega }=1$.

Although we assume that each scenario contains the information of the demands for all nodes and all periods, different assumptions could be done, being our approach still valid. For instance, it can also be assumed that there exist a different set of possible scenarios for the demands for each period and each region, or a set of possible scenarios for each period (independently of node or the region). For example, in this last case, for each $t\in T$ there would exist a set of scenarios $\Omega _t$. And for each $\omega \in \Omega _t$ we would have a vector of demands $d^{t\omega }=(d_1^{t\omega },\ldots , d_n^{t\omega })$, with an associate probability $p^{t\omega }$ with $\displaystyle \sum _{\omega \in \Omega _t}p^{t\omega } =1$.

With the above input information, our approach consists of determining an *optimal* redistribution planning for the equipment between units and a sharing policy by minimizing a function that takes into account the demand that is not able to be covered at each unit and period by the lack of resources.

## Mathematical programming model

In this section we provide a family of mathematical programming models to make decisions on: (1) the amount of product to be delivered between the different nodes of the network at each of the periods of the time horizon; and (2) the sharing of extra amounts received at each time period between the different groups $N_1, \ldots , N_p$, by minimizing different measures of the non-covered demand along the time horizon. Due to the existence of uncertainty in the demands, we propose robust solutions that perform *well* under any scenario. We describe the different mathematical programming approaches which differ in the measures of the non-covered demand that are considered but share the same sets of variables and constraints.

### Variables

We consider the following decision variables in our models.

- $x_{ij}^{t}$: amount of equipment to deliver from unit *i* to unit *j* at period *t*, for all $(i,j)\in W$, $t\in T$.
- $s_{ik}^{t}$: stock of equipment received by node *i* from the sharing of group $N_k$ at time period *t*, for all $i\in N_k$, $k\in P$, $t \in T$.

The above mentioned variables are identified with the two main decisions that are made by our models. The *x*-variables are identified with the distribution of existing equipment in the units in the given network while the *s*-variables model the distribution of new equipment incorporated to the network among the units in the different groups.

Observe that the above decision variables do not depend on the scenarios, since our aim is to provide a solution (distribution) with a good behavior under any of the scenarios that may occur.

We also consider the following auxiliary variables, which can be derived using the above decision variables, to ease the exposition of the models:

- $$y_{ij}^{t } = \left\{ \begin{array}{cl} 1 &{}\text {if at least one equipment is delivered from }i\text { to }j\text { at period }t,\\ 0 &{} \text{ otherwise, } \end{array}\right.$$, for all $(i,j)\in W$, $t\in T$. This variable allows us to control the different loads of equipment from a given unit. In particular $\displaystyle \sum _{j\in N: (i,j) \in W} y_{ij}$ is the overall number of loads that are prepared at period *t* from unit *i*, and will be upper bounded to avoid an excess of loads from each unit. Observe that this variables can be obtained from the *x*-variables as:
  $$\begin{aligned} y_{ij}^{t } = \left\{ \begin{array}{cl} 1 &{}\text {if }x_{ij}^t\ge 1,\\ 0 &{} \text{ otherwise } \end{array}\right. , \quad \text { for all } (i,j)\in W, t\in T. \end{aligned}$$
- $S_i^{t}$: accumulated stock in node *i* until period *t*:
  $$\begin{aligned} S_i^{t}=s_i^0 + \sum _{\begin{array}{c} k \in P: \\ i \in N_k \end{array}}\sum _{t^\prime \le t} s_{ik}^{t^\prime }, \quad \forall i \in N, t\in T. \end{aligned}$$
  It is the initial plus the extra stocks shared stock received by each of the groups where *i* belongs to.
- $R_i^{t}$: amount of product received until period *t* by unit *i* from other units:
  $$\begin{aligned} R_i^{t } = {\displaystyle \sum _{\begin{array}{c} j\in N:\\ (j,i)\in W \end{array}}} \displaystyle \sum _{\begin{array}{c} t^\prime \le t:\\ t^\prime + \ell _{ji}\le t \end{array}} x_{ji}^{t^\prime } , \quad \forall i \in N, t\in T. \end{aligned}$$
  It is the overall sum on all the units from what *i* is able to receive equipment and in all the periods $t^\prime$ in which the equipment are delivered plus the delivering time are previous or equal to *t*.
- $D_{i}^{t}$: amount of product delivered until period *t* by unit *i*:
  $$\begin{aligned} D_{i}^{t} = {\displaystyle \sum _{\begin{array}{c} j\in N:\\ (i,j)\in W \end{array}}}\displaystyle \sum _{t^\prime \le t} x_{ij}^{t^\prime }, \quad \forall i \in N, t\in T. \end{aligned}$$
- $H_i^{t \omega }$: effective excess at time period *t* from unit *i* under scenario $\omega \in \Omega$:
  $$\begin{aligned} H_i^{t \omega } = \max \{ 0, S_i^{t } + R_i^{t } - D_i^{(t-1) } -d_i^{t \omega } \}, \quad \forall \omega \in \Omega , i \in N, t\in T, \end{aligned}$$
  where $D_i^0 =0$. That is, the stock received until this period, plus the amount received until this period, minus the delivered until the previous period, minus the demand in this period. If the demand is not covered, $S_i^{t } + R_i^{t } - D_i^{(t-1)} -d_i^{t \omega }<0$, then the excess is 0. This amount represents the number of equipment that is available at the units in each period (under every scenario) after covering the demand at that unit. In order to linearly incorporate these variables to the model, we consider the auxiliary variable $\rho _{i}^{t\omega }$ that takes value 1 if $H_i^{t \omega } \ge S_i^{t } + R_i^{t } - D_i^{(t-1) } -d_i^{t \omega }$ and 0 otherwise. Then, the $H_i^{t \omega } = \max \{ 0, S_i^{t } + R_i^{t } - D_i^{(t-1) } -d_i^{t \omega } \}$ can be linearly formulated as:
  1 $$\begin{aligned} S_i^{t } + R_i^{t } - D_i^{(t-1) } -d_i^{t \omega } \ge U (\rho _{i}^{t\omega }-1),&\;\;\forall \omega \in \Omega , i\in N, t\in T, \end{aligned}$$
  2 $$\begin{aligned} S_i^{t } + R_i^{t } - D_i^{(t-1) } -d_i^{t \omega } \le U \rho _{i}^{t\omega },&\;\;\forall \omega \in \Omega , i\in N, t\in T, \end{aligned}$$
  3 $$\begin{aligned} S_i^{t } + R_i^{t } - D_i^{(t-1) } -d_i^{t \omega } - U(1-\rho _{i}^{t\omega }) \le H_i^{t \omega },&\;\;\forall \omega \in \Omega , i\in N, t\in T, \end{aligned}$$
  4 $$\begin{aligned} H_i^{t \omega } \le S_i^{t } + R_i^{t } - D_i^{(t-1) } -d_i^{t \omega } + U(1-\rho _{i}^{t\omega }),&\;\;\forall \omega \in \Omega , i\in N, t\in T, \end{aligned}$$
  5 $$\begin{aligned} 0 \le H_i^{t \omega } \le U \rho _{i}^{t\omega },&\;\;\forall \omega \in \Omega , i\in N, t\in T, \end{aligned}$$
  6 $$\begin{aligned} \rho _{i}^{t\omega } \in \{0,1\},&\;\;\forall \omega \in \Omega , i\in N, t\in T, \end{aligned}$$
  where *U* is a big enough constant. In particular, for each $\omega \in \Omega , i\in N, t\in T,$ *U* can be set as $U= U_i^{t\omega } = \max \{\sum _{i} s_i^0 + \sum _{t'\le t}\sum _{k}q_k^{t'},d_i^{t\omega }\}$. Constraints (1) and (2) allow to adequately define the value of $\rho _{i}^{t\omega }$ by the sign of the expression $S_i^{t } + R_i^{t } - D_i^{(t-1) } -d_i^{t \omega }$. In case $\rho _i^{t\omega }=1$, i.e., $S_i^{t } + R_i^{t } - D_i^{(t-1) } -d_i^{t \omega }\ge 0$, constraints (3) and (4) ensure that $H_i^{t\omega }$ takes value $S_i^{t } + R_i^{t } - D_i^{(t-1) } -d_i^{t \omega }$. Otherwise, by constraint (5) one is assured that $H_i^{t\omega }$ takes value zero.
- Non-Covered Demand of unit *i* at time period *t* under scenario $\omega \in \Omega$:
  $$\begin{aligned} NCD_i^{t \omega } = d_i^{t \omega } + D_i^{t } -S_i^{t } - R_i^{t }, \quad \forall \omega \in \Omega , i \in N, t\in T. \end{aligned}$$
  It is computed as the demand of the unit at that period plus the amount of equipment delivered from the unit at that period minus the accumulated stock and the product received until that period. In case $NCD_i^{t \omega } >0$, the demand plus the delivered are greater than the amount received, being not desirable and producing lack of resources at that period. Otherwise, if $NCD_i^{t \omega }<0$, the demand plus the number of deliveries is less than the received amount, then the demand $d_{i}^{t\omega }$, can be covered with the available equipment.
- Nonnegative Non-Covered Demand of point *i* at time period *t* under scenario $\omega \in \Omega$:
  $$\begin{aligned} {\overline{NCD}}_i^{t \omega } =\max \{0, {NCD}_i^{t \omega }\}, \quad \forall \omega \in \Omega , i \in N, t\in T. \end{aligned}$$
  As mentioned above, the actual demand that is not covered at a unit at a given time period under a given scenario is represented only when $NCD_i^{t \omega }>0$. Thus, this auxiliary variable consider only that positive part, in case it exists, and zero otherwise. These variables will be used in our objective functions instead of $NCD_i^{t \omega }$ to be somehow minimized, since the negative non-covered demand (which represents the positive stock) is not convenient to be minimized because it may provoke an excess of stock in the units. Taking into account that the ${\overline{NCD}}$-variables (or some of them) will be globally minimized in our objective functions, they can be easily incorporated to our mathematical programming formulations without using binary variables nor big-M constraints to obtain the optimal objective value for the problem. Concretely, they can be modeled as:
  $$\begin{aligned} {\overline{NCD}}_i^{t \omega }&\ge {NCD}_i^{t \omega },&\forall \omega \in \Omega , i \in N, t\in T,\\ {\overline{NCD}}_i^{t \omega }&\ge 0,&\forall \omega \in \Omega , i \in N, t\in T. \end{aligned}$$
  However, since in some of robust objective functions that we will consider, only the maximum of an aggregated function of these values is minimized, one is not assured that the values of the *x*- and the *s*-variables (which are the main decisions of our approaches) are adequately obtained but only those involved in the maximum values for the ${\overline{NCD}}$-variables. Thus, to obtain those values, one is required to use a similar linearization that the one used for the *H*-variables (constraints (1)–(6)) using binary variables and big-M constraints.

### Constraints

The above variables are related by means of a set of linear constraints that allows to represent adequately the reallocation and sharing problem under analysis:

1. The product to be delivered from a node, in each period and scenario, cannot exceed a percentage of the excess of that node: 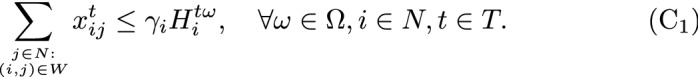 The constraint enforces that the overall amount of equipment delivered from *i* (under scenario $\omega$) in period *t* do not exceed the proportion of the stock that is allowed to be delivered from the unit to other units. It allows each unit to decide the proportion of the excess of equipment that is willing to deliver to other units. For risk-averse units, the proportion might be small, while for risk-adverse units, the proportion might be fixed to be large.
2. The amount to be delivered from a unit to other are zero unless the *y* variables take value one and viceversa. 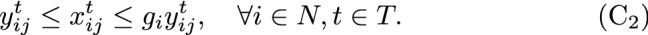 In case $y_{ij}^t=0$, then, one cannot deliver any product from unit *i* to *j*, otherwise and amount between 1 and $g_i$ can be sent.
3. Upper bound on the number of deliveries from a node to different nodes in each period: 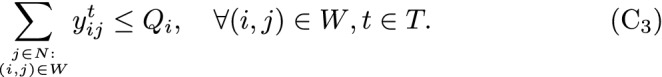 As mentioned in the definition of the *y*-variables, even in case a large stock is available in unit *i*, it is not realistic to assume that such a unit deliver equipment to many different units. This constraint limits such a number to $Q_i$.
4. Amount to be shared for each group and each period: 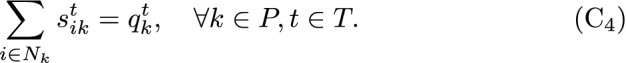 It is assumed that all the extra stock wants to be shared between the units in $N_k$. One may instead assume that not all the stock needs to be shared, and the constraint may be replaced by the same but with $\le$ instead the equation.
5. Avoid to simultaneously deliver and receive equipment in the same unit: 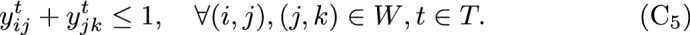 In particular, in case $k = i$, the constraint enforces that no bidirectional deliveries are allowed at the same period. Note that one may replace the set *W* by a subset of it to allow some of the units to deliver and receive at the same period.
6. Upper bound on the storage capacity of each hospital and each period: 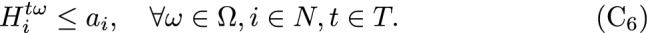 Most units may not have unlimited space to store all the equipment they receive. This constraints avoid this effects and allows receiving material only if they have space to store it or directly use it.

### Objective functions

Our stochastic mathematical programming models will have the following common shape:

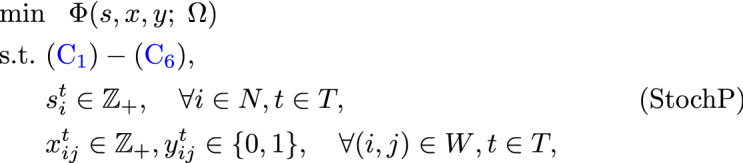



where $\Phi (\cdot )$ will be a measure of the overall non-covered demands, and will determine the difference between the distinct approaches.

We consider four different robust objective functions for the mathematical programming problem described above to be minimized:

- *Maximum non-covered expected demand of the units within the time horizon*:
  $$\begin{aligned} \Phi _1(s,x,y; \Omega ) = \max _{i \in N} \displaystyle \sum _{\omega \in \Omega } p^\omega \displaystyle \sum _{t\in T} {\overline{NCD}}_i^{t\omega }. \end{aligned}$$
  This function equilibrates the expected non-covered demand for all the units along the whole time horizon.
- *Maximum non-covered expected demand of the demand points at each period*:
  $$\begin{aligned} \Phi _2(s,x,y; \Omega ) = \max _{i \in N} \max _{t\in T} \displaystyle \sum _{\omega \in \Omega } p^\omega {\overline{NCD}}_i^{t\omega }. \end{aligned}$$
  Here, one equilibrates, not only units for the whole time horizon, but also the different periods, avoiding tiny non-covered demands in a period at the price of large non-covered demands in others.
- *Maximum non-covered expected demand in each region within the time horizon*:
  $$\begin{aligned} \Phi _3(s,x,y; \Omega ) = \max _{k=1, \ldots , L} \displaystyle \sum _{\omega \in \Omega } p^\omega \displaystyle \sum _{i \in M_k} \displaystyle \sum _{t\in T} {\overline{NCD}}_i^{t\omega }. \end{aligned}$$
  where $M_1, \ldots , M_L \subset N$ is disjoint partition of *N* in *L* sets. Instead of finding fair solutions for all units and periods, in $\Phi _3$, the units are aggregated by these regions, being the criterion to find equilibrate regional solutions. These sets represent different non-overlapping regions in which the effect of a distribution planning wants to be measured. In practice, they can be determined by the political borders of a country (states, regions, districts, etc) in which the policies stablished at each of them want to be evaluated.
- *Minimize the total non-covered expected demand*:
  $$\begin{aligned} \Phi _4(s,x,y; \Omega ) = \displaystyle \sum _{\omega \in \Omega } p^\omega \displaystyle \sum _{i \in N} \displaystyle \sum _{t\in T} \ {\overline{NCD}}_i^{t\omega }. \end{aligned}$$
  Finally, this function account for the overall non-covered demand for all scenarios, units and period along the time horizon.

The specific shape of the objective functions, among those described above, that is used in our decision tool must be chosen by the decision maker based on its own preferences. $\Phi _1$ allows one to find fair solutions by units (e.g., hospitals) taking into account the most harmed ones, in terms of the non covered demand, while in $\Phi _3$, the fairness is measured by regions instead of single units. Objective $\Phi _2$ also accounts for equilibrating the non covered demand by periods, avoiding low non covered demand in some periods at the price of high non covered demands in others. Finally, $\Phi _4$ is the classical averaged measure which allows to globally minimize the non covered demand, which may harm some units to benefit others. The decision maker may also run all the models and, in view of the results, decide the most reasonable situation.

Apart from the four objective functions $\Phi _1, \ldots , \Phi _4$, we also consider in our approach their max-regret counterparts. For any $\omega \in \Omega$ we denote by $\Phi ^*(\omega )$ the optimal value of the problem above but only under scenario $\omega \in \Omega$, i.e.,

$$\begin{aligned} \Phi ^*(\omega ) := \min&\,\,\, \Phi (s,x,y; \{\omega \})\\ \text{ s.t. }&(\mathrm{C}_{1})-(\mathrm{C}_{6}),\\&s_i^{t} \in \mathbb {Z}_+,\quad \forall i \in N, t \in T,\\&x_{ij}^{t} \in \mathbb {Z}_+, y_{ij}^{t} \in \{0,1\}, \quad \forall {(i,j)\in W}, t \in T, \end{aligned}$$

for any of the objective functions defined above ($\Phi \in \{\Phi _1, \Phi _2,\Phi _3,\Phi _4\}$).

We define the regret of a solution (*x*, *y*, *s*) under scenario $\omega \in \Omega$ as:

$$\begin{aligned} \Phi ^\mathrm{Regret}(s,x,y; \omega ) = \Phi (s,x,y;\{\omega \}) - \Phi ^*(\omega ), \end{aligned}$$

that is, the difference between the actual evaluation in the global objective function $\Phi \in \{\Phi _1, \Phi _2,\Phi _3,\Phi _4\}$ of feasible solution (*s*, *x*, *y*) under scenario $\omega$ and the optimal value obtained for such a single scenario $\omega$.

The minmax regret criterion seeks a solution minimizing the maximum regret among all scenarios, that is, it seeks a solution whose value is as close as possible to the optimal value for every scenario (see, e.g. Aissi et al. (2009); Ben-Tal et al. (2009); Kasperski (2008); Kouvelis and Yu (1997) and the references therein). The regret version of (${\mathrm{StochP}}$) is:

$$\begin{aligned} \displaystyle \min \; \max _{\omega \in \Omega }&\;\;\; \Phi ^\mathrm{Regret}(s,x,y; \omega )\nonumber \\ \text{ s.t. }&({\mathrm{C}}_{1})-({\mathrm{C}}_{6}),\\&s_i^{t} \in \mathbb {Z}_+,\quad \forall i \in N, t \in T,\\&x_{ij}^{t} \in \mathbb {Z}_+, y_{ij}^{t} \in \{0,1\}, \quad \forall {(i,j)\in W}, t \in T. \end{aligned}$$

The above formulation can be equivalently rewritten as:

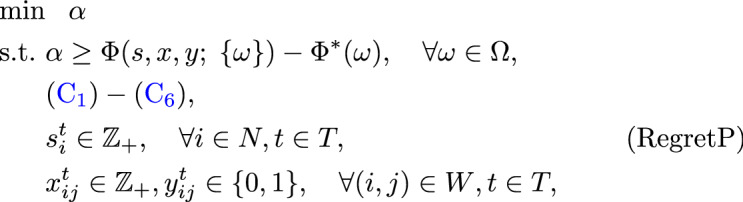



for each $\Phi \in \{\Phi _1, \Phi _2,\Phi _3,\Phi _4\}$, resulting in four alternative objective functions $\Phi _1^\mathrm{Regret}$, $\Phi _2^\mathrm{Regret}$, $\Phi _3^\mathrm{Regret}$, $\Phi _4^\mathrm{Regret}$.

## Math-Heuristic procedure

The mathematical programming formulations described in Sect. 3 involve integer variables to represent the decision variables concerning the deliveries (*x*) and the shared amounts (*s*). Furthermore, they use other sets of auxiliary binary and continuous variables in order to adequately represent the constraints and the objective functions, as *y* or those that allow to model the excess of stock or the nonnegative non-covered demand. Thus, the Mixed Integer Linear Programming (MILP) model becomes hard to solve when the number of units (*N*), periods (*T*) and scenarios ($\Omega$) is large, as usual. Actually, using the real-data of the COVID-19 case in Spain that we analyze in Sect. 5 ($\vert N\vert =106$, $\vert T\vert =49$ and $\vert \Omega \vert =3$), the optimization solver (Gurobi) was not even able to load the model in 12 hours.

In this section, we describe a math-heuristic approach that allows us to obtain good quality feasible solutions for the problem in reasonable computational times, but still using mathematical programming tools to solve up to optimality some subproblems. The main idea under the heuristic is to split the time horizon in shorter time horizons and *merge* the obtained results of the smaller problems adequately.

In our approach we split $T=\{1, \ldots , q\}$ into smaller sorted non-overlapping subperiods, $T_1, \ldots , T_K$ with $T_k = \{t_{k-1}, t_{k-1}+1, \ldots , t_{k}-1\}$ for $k=1, \ldots , K$, where $1=:t_{0}< t_{1}< \cdots < t_{K}:=q$. Although one may solve the problem at each of the sets $T_k$ instead of the whole *T*, the obtained solution is not feasible for our problem since the initial stock at the beginning of each subperiod is not defined, except for the first interval. To overcome this difficulty we propose an approach to adequately *glue* the obtained solutions to construct a feasible solution of the original problem. For the sake of this gluing process, instead of solving the problems within the time periods $T_1, \ldots , T_K$, we consider the subperiods $T_1^+, \ldots , T_K^+$, where $T_k^+ = T_k \cup \{t_k\}$, i.e., each subperiod is linked with the next subperiod throught a single time instant. Next, the MILP is solved for $T_1^+$, calculating the reallocation and sharing policies for that interval, for all its time periods instead those of the last one, $t_1$, where, instead, we compute the excess of each unit at that period. This excess is used as input of the initial stock for solving the next subperiod, $T_2^+$. The process is repeated until the complete execution of all the subperiods is performed. Observe that, unless delivering times are zero, the *x* and *y* variables in the last period of each interval are zero, since it is not possible to cover any demand. The values of the *x* and *s*-variables are sequentially obtained, while the value of the objective function (for each of them) has to be constructed once the procedure is terminated. In Algorithm 1 we show the pseudocode of proposed procedure.

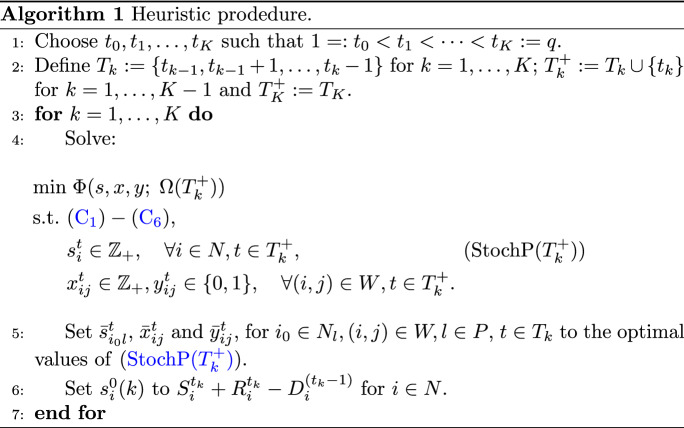



### Proposition 1

Algorithm 1 provides a feasible solution for (${\mathrm{StochP}}$).

### Proof

Observe that at each time period, *t*, the obtained solution verifies all the constraints of the model, except ($\mathrm{C}_1$) and ($\mathrm{C}_6$) which depend on the auxiliary *H*-variables, since they are separable by the index *t*. For the case of ($\mathrm{C}_1$) and ($\mathrm{C}_6$), they depend on the *H*-variables which were defined as:

$$\begin{aligned} H_i^{t \omega } = \max \{ 0, S_i^{t } + R_i^{t } - D_i^{(t-1) } -d_i^{t \omega } \}. \end{aligned}$$

Thus, they depend on the amount of product received and delivered until the previous period, and also on the stock accumulated until that period (which are accumulated from the initial period to period *t*). Let us denote by:

$$\begin{aligned} h_i^{t}(k) =S_i^{t}(k) + R_i^{t }(k) - D_i^{(t-1) }(k), \;\;\text {for all } i \in N, \omega \in \Omega , \end{aligned}$$

the amount of available product in unit *i* at period $t\in T_k$ before attending the demand of the unit, but after receiving (from other units or shared by its region) equipment and delivering, where

$$\begin{aligned} S_i^{t}(k)&= s_i^0(k) + \displaystyle \sum _{\begin{array}{c} j\in P:\\ i \in N_j \end{array}} \displaystyle \sum _{t^\prime \le t} s_{ij}^{t^\prime },\\ R_i^{t }(k)&= {\displaystyle \sum _{\begin{array}{c} j\in N:\\ (j,i)\in W \end{array}}} \displaystyle \sum _{\begin{array}{c} t_{k-1} \le t^\prime \le t:\\ t^\prime + \ell _{ji}\le t \end{array}} x_{ji}^{t^\prime },\\ D_{i}^{t}(k)&= {\displaystyle \sum _{\begin{array}{c} j\in N:\\ (i,j)\in W \end{array}}}\displaystyle \sum _{t_{k-1}\le t^\prime \le t} x_{ij}^{t^\prime }, \end{aligned}$$

for $$k \in \{1, \ldots , K\}$$, $$i\in N$$ and $$t \in T_k$$.

Note that this values are the subperiod counterparts of the global variables defined in our model.

For $t \in T_k$ we get that:

$$\begin{aligned} \begin{aligned} h_i^{t}(k)&= S_i^{t_k} + R_i^{t_k} - D_i^{(t_k-1) } + \sum _{\begin{array}{c} j\in P:\\ i \in N_j \end{array}} \displaystyle \sum _{t^\prime \le t} s_{ij}^{t^\prime } + {\displaystyle \sum _{\begin{array}{c} j\in N:\\ (j,i)\in W \end{array}}} \displaystyle \sum _{\begin{array}{c} t_{k-1} \le t^\prime \le t:\\ t^\prime + \ell _{ji}\le t \end{array}} x_{ji}^{t^\prime } - {\displaystyle \sum _{\begin{array}{c} j\in N:\\ (i,j)\in W \end{array}}}\displaystyle \sum _{t_{k-1}\le t^\prime \le t-1} x_{ij}^{t^\prime }\\&= S_i^t + R_i^t - D_i^t. \end{aligned} \end{aligned}$$

Thus, $H_i^{t\omega } = \max \{0, h_i^{t}(k)-d_i^{t\omega }\}$ if $t \in T_k$. Hence, the *H*-variables in our model are adequately recovered from the solutions obtained solving the problem by subperiods. Since for each of the subproblems on $T_k^+$, the constraints:

$$\begin{aligned} \displaystyle \sum _{\begin{array}{c} j\in N:\\ (i,j)\in W \end{array}} x_{ij}^{t} \le \gamma _i \max \{0, h_i^t(k)-d_i^{t\omega }\} \text { and } H_i^{t \omega }\le a_i \end{aligned}$$

are verified for $t \in T_k$ and $i \in N$, $\omega \in \Omega$ and $k \in \{1, \ldots , K\}$, then ($\mathrm{C}_1$) and ($\mathrm{C}_6$) are also verified. $\square$

From the above result, we get that our procedure provides a feasible solution to our problem, and then gives us upper bounds for the exact optimal values of our problems.

## Case study: reallocation and sharing of ventilators in Spain

One of the main causes for the critical situation in hospitals during the COVID-19 crisis in Spain was, like in most of the countries, the high demand of invasive mechanical ventilation among severe patients, together with the lack of this resource in some hospitals around the country. Invasive mechanical ventilation is used to assist patients with serious breathing problems (Tobin 2001; NHLBI n.d.).

We devote this section to analyze the reallocation and sharing of invasive mechanical ventilators (from now on ventilators) in two regions in Spain with different demand distribution during the first wave: the region of Madrid, in which the pandemic caused a large amount of critical patients and dead, and the region of Andalucía, in which the situation was slightly less critical. We use the proposed mathematical programming models to determine if the covered demand of patients needing a ventilator could have been significantly improved if reallocation and sharing would have been applied during the first COVID-19 wave, the 49 days from March 8th to April 25th, 2020.

Even thought we focus in the reallocation of ventilators, as already mentioned, any other type of health equipment could be considered for sharing and allocation.

### Input information

In what follows we describe the input information that we use in our models as well as the results obtained after running them. All the input information that we use in our experiments are available in the GitHub repository https://github.com/vblancoOR/RedistributionCOVID19.

### Graph structure

We consider two types of graph structures trying to simulate possible real networks of the regions of Madrid and Andalucía. While the region of Madrid has 51 hospitals, the region of Andalucía has 106.

**Fig. 2:**
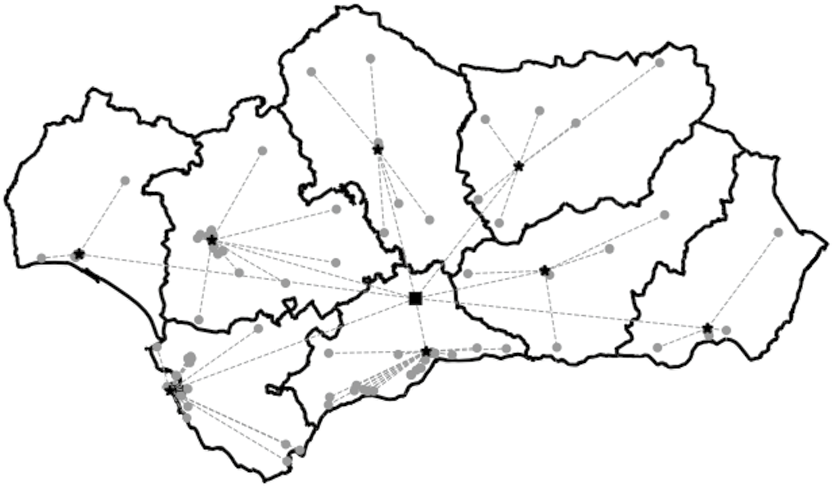
Graph structure with logistics centers in Andalucía

Note that being the graph structure an input, it can be modified to adjust the reality of the regions. In particular, we consider two types of graphs in our experiments:

- *Complete (C)*: We consider a complete graph in which all nodes (units) are connected bidirectionally.
- *Logistic Centers (LC)*: We incorporate logistic centers of provinces and regions, and we consider that each hospital of a province is only bidirectionally linked with the logistic center of the province, and the logistic centers of the provinces are linked through the regional logistic center. For each of the two considered regions the situation is different:
  - *R*egion of Madrid: Since this region has a single province, we assume that the unique logistic center is located in the city of Madrid in *Hospital de la Paz*.
  - *R*egion of Andalucía: In this region there exist a logistic center in each of its eight provinces. We assume that the regional logistic center is located in *Hospital de Antequera* in Málaga (geographical center of Andalucía). This graph has 105 arcs. In Fig. 2, we show, this graph structure in this region.

For the region of Madrid a single $N_1$ region is considered (containing the 51 hospitals in the region). In Andalucía, we consider $N_1, \ldots , N_8$ as the sets containing each of the hospitals in the different provinces. The sets *M* for the objective functions $\Phi _3$ and $\Phi ^\mathrm{Regret}_3$ coincide with the *N*-sets, that is, the regions for which fair solutions are desired are the same as those for which the sharing policies are designed.

Furthermore, in Andalucía, for the LC-graph structure, we also use the set $N_9$ containing the eight logistic centers (but it is not included in the *M*-sets used in the $\Phi _3$-objective functions).

### Initial stocks

The initial number of invasive mechanical ventilators in each hospital is crucial to conduct an accurate study. However, these official stocks were not publicly available in Spain. In order to estimate them we collected information from different sources. Since the main reason for hospitalizing a patient in an ICU is to provide ventilatory support (Tobin 2001), we assume that this number coincides with the number of ICU beds. Note that these amounts may be slightly larger due to extra ventilators situated at other types of beds in some hospitals. This underestimation may be favorable since those ventilators are available for the hospitals in case more patients than the estimated need a ventilator. The proportion of ICU beds in public and private hospitals of each region (Datadista n.d.) together with the number of beds in each hospital (Catálogo Nacional de Hospitales 2019) allows us to estimate the initial stock of ventilators of each hospital.

### Extra stock

The extra stock at each time period indicates the new available ventilators to share (if any) in that period among the hospitals of a given region. During the COVID-19 crisis different national or regional governments have bought and received extra invasive mechanical ventilators as reported by some national newspapers (Libertad Digital 2020; Hoy 2020; El Médico Interactivo 2020; Libre Mercado 2020; El País 2020; El Periódico 2020; RTVE 2020among many others). The situation in the two considered regions is different:

- *Region of Madrid:* In this region, the regional government received 351 ventilators at the end of March and 213 ventilators at the early April (El Periódico 2020). It also received ventilators from other regions of Spain: Galicia lent 11 ventilators on March 27th, Andalucía 22 ventilators at the end of March, Extremadura 10 ventilators at the end of March and Murcia 9 ventilators in early April (El País 2020; Libertad Digital 2020; Hoy 2020; El Médico Interactivo 2020).
- *Region of Andalucía:* In Andalucía, there is no public information about the extra stock. However, a significant issue in this region is that Andalucía started to manufacture its own ventilators under the project *Andalucía Respira* (Andalusia Government 2020; El País 2020). Although these ventilators fulfill the quality requirements established by the Ministry of Health, we did not find any information stating that they have been distributed by the region yet.

Apart from the above, some extra stock was provided from the Spanish Government and private donations. A total of 2400 ventilators was received to share among all the regions (Libre Mercado 2020; RTVE 2020); but there is no information about when and where the ventilators were allocated. We assume that these ventilators were distributed among the regions by means of population. Hence, in the cases of Madrid and Andalucía, we estimate that they received, in the second week of April, 340 and 429 ventilators, respectively.

### Capacities

- $Q_i$: The maximum number of deliveries from a hospital depends on the graph structure. For the C-Graph we set it to 5, to avoid extra work on preparing packages of ventilators for different trucks. For LC-graph, the parameter for the logistic centers was fixed to infinity, while for the hospitals, we set it to 0.4 times the number of adjacent nodes to the logistic center.
- $\gamma _i$: The percentage of excess that can be delivered from a hospital was fixed to 0.8.
- $g_i$: The amount of ventilators to be delivered by each hospitals is set to 20, as a measure of transport capacity.
- $a_i$: The storage limit for the hospitals is set to twice the number of ICU beds in the hospital. For logistic centers the parameter is set to infinity.

### Shipping times

The arc weights of the graphs are defined based on real geographical distance between the nodes. We compute the shortest paths between each pair of nodes (in each graph structure) considering real geographical distance and then, we translate them to travel times, assuming that the largest distance is traversed in one day. The remaining travel times are computed proportionally to this largest travel time of one day. Apart of that, we include in the shipping times different graph-dependent processing times: one day for the C-graph and 0.1 days per used logistic center in the LC-graph.

### Demands

We estimated the daily ventilator demands based on real ICU demands of COVID-19 patients in Madrid and Andalucía, published by the Spanish and Andalucía governments from 08/03/2020 to 25/04/2020 (first COVID-19 wave data) (COVID19. Spain dataset n.d.; COVID19. Andalusian dataset n.d.).

This data was collected differently in each region. The region of Madrid reported the daily ICU demands, and then, ready to be incorporated to the models. On the other hand, the government of Andalucía reported the accumulated demand of ICU. Tons of studies have been published during the pandemic all around the world in which different estimations of the demand were proposed with different statistical methodologies (see e.g., Benítez-Peña et al. (2021); Garcia-Vicuña et al. (2022); Mahmoudi et al. (2020, 2021, 2021); Maleki et al. (2020, 2020), among many others). We adopt a simplified estimation of the demand. We estimated the daily demands as follows: for each hospital, we compute the daily number or new COVID-19 patients in ICU and we assume that each of them stays in ICU 21 days (the average number of days the patients stay on a ICU bed during the first wave (RTVE 2020)). Scenario “Real” was created with these estimated demands. This scenario can be considered as the closest to the real situation that Spain lived during the first wave.

Using these demands, we randomly generate two more scenarios as follows:

- Choose $r_j^-, r_j^+$ uniformly distributed in [0, 0.5], for each province $j \in P$.
- Choose ${\tilde{r}}_i^-$ (resp. ${\tilde{r}}_i^+$) uniformly distributed in $[0,r_j^-]$ (resp. $[0,r_j^+]$), for each hospital *i* in the province, *j*, and set, for each scenario the following demands:
  - “Pessimistic” Scenario : $${\tilde{d}}_i^t =(1+ {\tilde{r}}_i^+) d_i^t, \quad i\in N_j, j \in P, t\in T.$$
  - “Optimistic” Scenario: $${\tilde{d}}_i^t = (1- {\tilde{r}}_i^-) d_i^t, \quad i\in N_j, j \in P, t\in T.$$
  In case ${\tilde{d}}_i^t = 0$ we set random integer value in $\{1,2\}$. We assume that the three scenarios are equally likely.

**Fig. 3:**
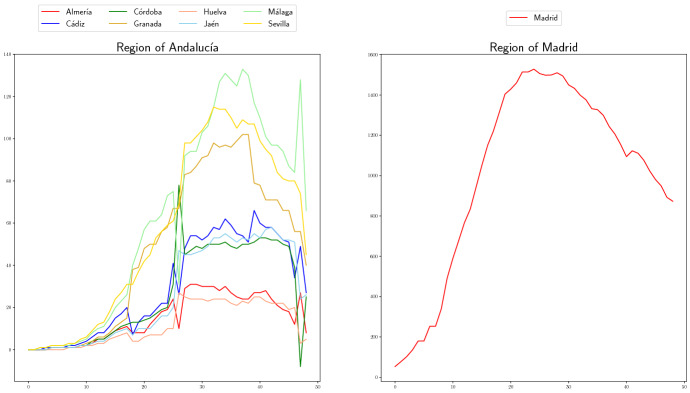
Real demands of ventilators for Region of Andalucía (left) and Region of Madrid (right)

In Fig. 3, we show the demands on Scenario Real for the two regions.

### Results

The models have been coded in Python 3.7 and using as optimization solver Gurobi 9.0 in a MacBook Pro with a Core 2 Duo CPU clocked at 2,66 GHz and 4GB of RAM memory. We have run our math-heuristic algorithm partitioning the time horizon into 12 subperiods. A time limit of 1 hour was fixed for solving the subproblems, although none of our models reached such a limit.

We have applied our approach to the regions of Madrid and Andalucía. Moreover, we have also run the models in case no sharing is allowed between the hospital. The aim is to compare the obtained redistribution and sharing policies to the real situation in Spain, in which redistribution was not implemented. However, since there is no information about the sharing policies of the extra stock in the real situation, we assume that it was performed *optimally* according to our models (only fixing in them to 0 the *x*-variables). Note that we compare our redistribution and sharing proposal to a situation which is better than the actually implemented.

In the following sections, we analyze the results and conclusions obtained through the numerical study. We illustrate them with different graphics and figures for particular configurations of scenarios, objective functions or types of graphs. However, the rest of the figures for the remaining configurations of scenarios, functions or graphs can be found in https://github.com/vblancoOR/RedistributionCOVID19 for the interested readers.

#### Redistribution vs. no redistribution

**Fig. 4:**
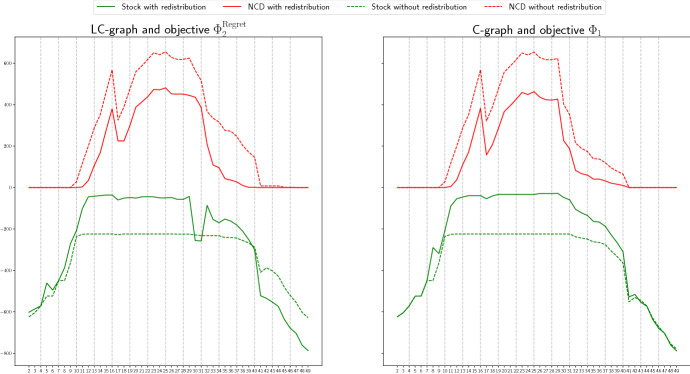
Non-covered demand (red lines) and available stock (green lines) at each time period, if the Real scenario occurs, with (continuous line) and without (dashed line) redistribution, in Madrid, for LC-graph and objective $\Phi _2^\mathrm{Regret}$ (left) and for C-graph and objective $\Phi _1$(right) (color figure online)

**Fig. 5:**
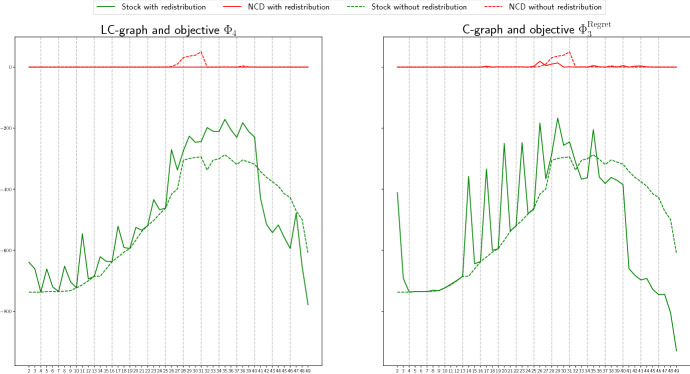
Non-covered demand (red lines) and available stock (green lines) at each time period, if the Real scenario occurs, with (continuous line) and without (dashed line) redistribution, in Andalucía, for LC-graph and objective function $\Phi _4$ (left) and for C-graph and objective function $\Phi _3^\mathrm{Regret}$(right) (color figure online)

We start by comparing the non-covered demand, that is, the number of patients needing a ventilator that were not attended due to the lack of this resource, if the proposed redistribution is carried out, or not. In Figs. 4 and 5, in each of the pictures, the continuous red line represents the total non-covered demand at each time period if the Real scenario occurs and redistribution is allowed. The dashed red line shows the total non-covered demand at each time period, if the Real scenario happens and only the redistribution of the extra stock is allowed, but not the sharing of available stock. Remind that this second case represents a situation better than what was actually applied in Spain, since in this case, the redistribution of the extra stock is done optimally. For simplicity, we will refer to this second case as the *without redistribution* case. The continuous and dashed green lines represent, respectively, the total available stock in the two described situations: with or without redistribution. Notice that we solve the models taking into account that any of the three considered scenarios can occur, but we represent in these figures the actual behavior if the obtained solution is implemented when the Real scenario happens.

We show in Fig. 4 the case of the Madrid region, for the graph with logistic centers and objective $\Phi _2^\mathrm{Regret}$ in the left picture, and for the complete graph and objective $\Phi _1$ in the right one. We can observe that in all the cases, when redistribution is not carried out, there exist always available stock but also demand that is not covered. For instance, in the second picture we see that in period 25, there are around 250 available ventilators but around 650 patients that are not attended. However, when redistribution is considered, in the periods in which the non-covered demand is positive, the available stock is almost zero, that is, the available stock is redistributed and used. This implies a significant decrease in the number of non-treated patients. For instance, in the same case described before, the non-covered demand reduces to less that 470. A similar behaviour can be observed in the rest of objectives, graphs and scenarios.

In Fig. 5, we show the case of the Andalucía region, for a graph with logistic centers and objective $\Phi _4$ (right), and for the complete graph and objective function $\Phi _3^\mathrm{Regret}$ (left). In this case, we can observe that most of the demand is covered in both cases, with and without redistribution. The reason for this is the availability of stock to cover all the demand in most of the periods. However, there is a critical period, between $t=25$ and $t=33$, in which the demand increases (see Fig. 3) and the non-covered demand is positive for the case without redistribution, even thought there exist more than 200 available ventilators. It can also be observed that for some objective functions, the non-covered demand in the redistribution case is also positive despite existing available stock. The reason for this can be twofold: 1) Since the problem is solved using the heuristic described in Algorithm 1 in which the total time period is split into smaller periods, the heuristic is not able to anticipate, for all the objective functions, the rapid increase of the demand after period 24; 2) the collected information for the provinces of Málaga and Sevilla is not accurate, with a sudden big increase and decrease in period 27, as can be observed in Fig. 3, which can not always be efficiently handled. However, in most of the cases, the total non-covered demand is lower for the redistribution case, being this value even zero for some of the objective functions, as it is the case of $\Phi _4$.

We conclude therefore that redistributing the available stock significantly increases the number of treated patients.

#### Comparison of redistribution and sharing policies

We compare in this section the behavior of the redistribution through the time horizon and provinces.

**Fig. 6:**
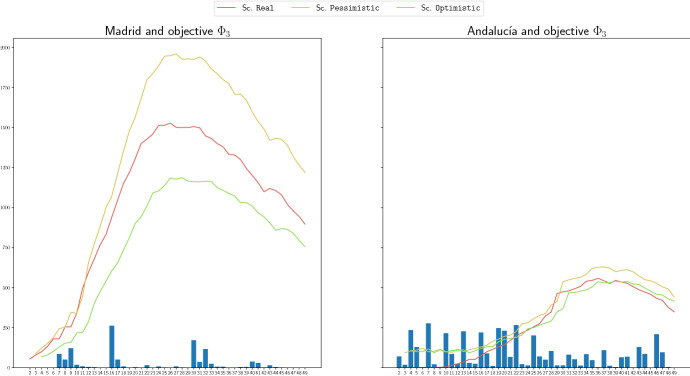
Amount of redistributed stock throught the LC-graphs, at each time period, for the objective function $\Phi _3$, for the region of Madrid (left) and the region of Andalucía (right)

**Fig. 7:**
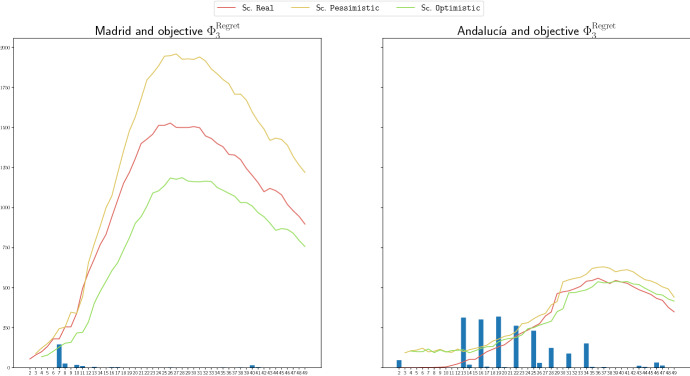
Amount of redistributed stock throught the C-graphs, at each time period, for the objective function $\Phi _3^\mathrm{Regret}$, for the region of Madrid (left) and the region of Andalucía (right)

We show in Fig. 6, the number of redistributed ventilators through the LC-graphs in the achieved solution, at each time period, for the region of Madrid (left) and Andalucía (right) for objective function $\Phi _3$. We also include in such graphics the demand curves for the three scenarios. Figure 7 shows the same but for the case of C-graphs and objective function $\Phi _3^\mathrm{Regret}$. We can observe that the amount of redistributed stock is much higher in the region of Andalucía than in the region of Madrid. This is caused by the fact that the number of demanded ventilators in Madrid is much greater than such number in Andalucía. The high demand in most of the hospitals in Madrid make practically nonexistent the availability of stock to share. The existence of stock to redistribute in periods 6 to 10 in Madrid is due to the lower demand, and the availability of stock in periods 16, 30 and 32 in Madrid in Fig. 6 is due to the entrance of a high quantity of extra stock: 351, 213 and 115 ventilators, respectively, to the logistic center that it is later redistributed. This effect is not observed in the case of the C-graphs, Fig. 7, because here the extra stock is directly distributed among the hospitals, and in this graphic we only show the redistribution among hospitals, not the distribution of the extra stock. In the case of Andalucía, the highest amounts of shared ventilators coincide with the periods in which there is lower demand, and therefore more available stock to redistribute anticipating future increases in the demands. In the case of the graphs including logistic centers, there exist more periods with redistribution due to the same effect explained for the case of Madrid, and also to the fact that the delivery constraints imposed to the logistic center are less restrictive than for the hospitals.

Note that we are optimizing the non-covered demand, and hence, the management of the available stock could have been done better.

**Fig. 8:**
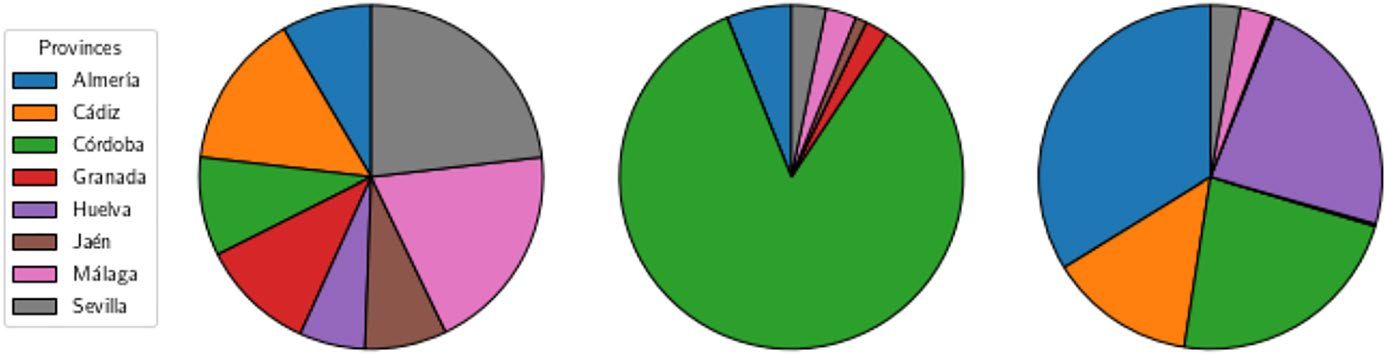
Distribution of proportion of extra stock in the provinces of Andalucía: by population (left), by our model for LC-graph and $\Phi _4$ (center), and C-graph and $\Phi _{4}^\mathrm{Regret}$(right)

We include in the following Fig. 8, three different pie charts. The first one contains the distribution by proportion of population of the regions in Andalucía. In the remaining charts, we show the proportion of extra stock in the obtained solution for the model with redistribution and objective functions $\Phi _4$, and $\Phi _4^\mathrm{Regret}$, respectively, for LC-graphs in the first case, and C-graphs in the last case. Note that the share of extra stock is performed in our model based on the demand required by each hospital and not on population, and then, the pie charts reflect that our model allocates the extra stock by demand. We can observe for instance in the diagram for $\Phi _4$ and LC-graphs, the one in the center, that most of the extra stock is allocated in the province of Córdoba, which is not concentrating the highest proportions of population. For the case of the C-graph and objective function $\Phi _4^\mathrm{Regret}$, the distribution is not mostly concentrated in a single province, it is withal more divided among different provinces. However, also in this case, the distribution is not carried out according to the proportion of inhabitants. For instance, we can see that despite being Sevilla the province with the highest proportion of inhabitants in Andalucía, the proportion of extra stock sent to this province (grey) is one of the lowest ones.

In these pie charts we can also observed the previously described effect: the extra stock is more distributed into different provinces in the case of the C-graphs than in the case of the LC-graphs. This is due to the fact that it is easier to redistribute later the available stock from the logistic centers than from the hospitals, and also to the fact that the logistic centers have a higher capacity to store stock.

#### Comparison of non-covered demand by scenarios

**Fig. 9:**
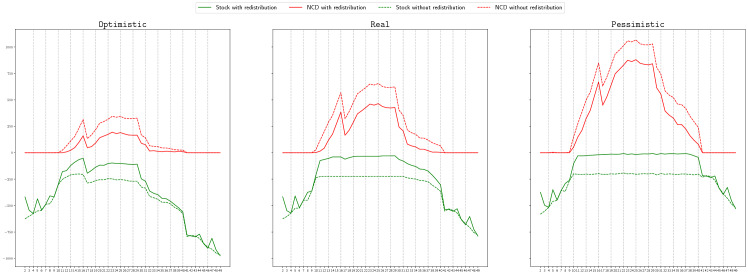
Non-covered demand (red lines) and available stock (green lines) at each time period, if the Optimistic (left), Real (center) or Pessimistic (right) scenario occurs, with (continuous line) and without (dashed line) redistribution, in Madrid, for C-graph and objective function $\Phi _1^\mathrm{Regret}$ (color figure online)

**Fig. 10:**
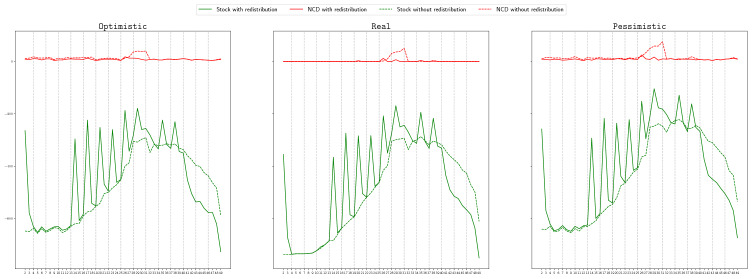
Non-covered demand (red lines) and available stock (green lines) at each time period, if the Optimistic (left), Real (center) or Pessimistic (right) scenario occurs, with (continuous line) and without (dashed line) redistribution, in Andalucía, for C-graph and objective function $\Phi _4$ (color figure online)

We show in this section, in Figs. 9 and 10, the behaviour of the non-covered demand if the achieved solution is implemented in each of the three considered scenarios: optimistic (left), real (center) and pessimistic (right). Each graphic in each of these figures follows the same style that the graphics presented in Fig. 4. Figure 9 illustrates the case of Madrid and objective function $\Phi _1^\mathrm{Regret}$, and Fig. 10 the case of Andalucía and objective function $\Phi _4$. In both cases we can appreciate that the pattern of the non-covered demand is practically the same for the three different scenarios, since the tendency of the demand is the same in the three scenarios, but, as expected, the higher the amount of demand, the higher the non-covered demand. For instance, in the worst moment in Madrid and our approach is applied, if optimistic scenario occurs, the non-covered demand is around 250, meanwhile if pessimistic scenario occurs, the non-covered demand is more than 750. For the case without redistribution, these amounts are around 300 and more than 1000.

#### Complete graph vs. logistic center graph

**Fig. 11:**
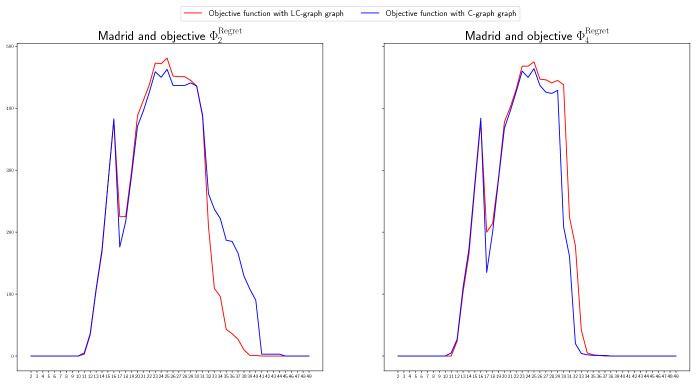
Non-covered demand in Madrid at each time period for the LC-graph (red) and C-graph (blue), for objective functions $\Phi _2^\mathrm{Regret}$ (left) and $\Phi _4^\mathrm{Regret}$ (right) if the Real scenario occurs when considereing redistribution (color figure online)

**Fig. 12:**
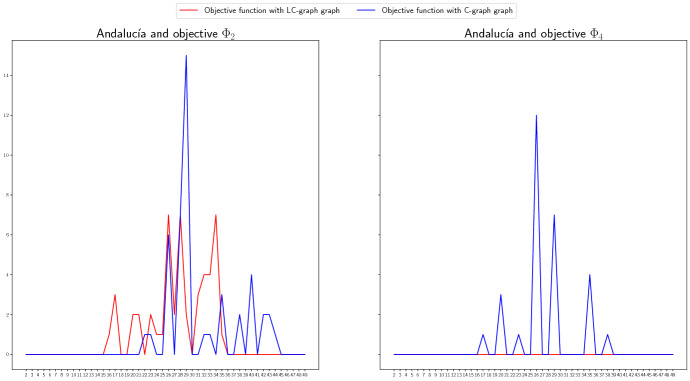
Non-covered demand in Andalucía at each time period for the LC-graph (red) and C-graph (blue), for objective functions $\Phi _2$ (left) and $\Phi _4$ (right), if the Real scenario occurs when considereing redistribution (color figure online)

We include in this section a comparison of the non-covered demand for the LC- and C-graphs. In Fig. 11, we do it for the case of Madrid and objective functions $\Phi _2^\mathrm{Regret}$ and $\Phi _4^\mathrm{Regret}$, and in Fig. 12 for the case of Andalucía and objective functions $\Phi _2$ and $\Phi _4$. The red line represents the non-covered demand for the LC-graphs, and the blue line for the C-graphs. For the case of Madrid, the behavior under both graph structures is quite similar. Using logistic centers to redistribute the ventilators seems to perform a bit worse than not using them for more cases (according to the settings we used in the numerical study), but not for all of them. This could be due to the need of considering more than one logistic center since the demand is very high. For the case of Andalucía, using logistic centers seems to perform better and to lead to less high demand peaks. For example, when minimizing the total non-covered expected demand, that is, for objective function $\Phi _4$ (right graphic in Fig. 12), using logistic centers results much better than not using them.

#### Different criteria

**Fig. 13:**
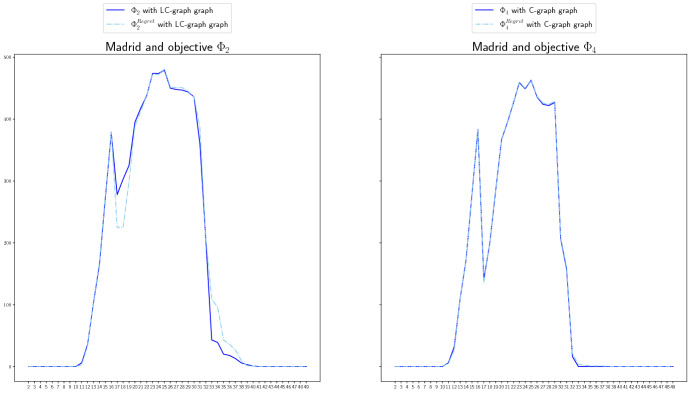
Non-covered demand in Madrid, at each time period, for the regret (dashed line) and not regret (continuous line) versions of objective functions $\Phi _2$ (left) and $\Phi _4$ (right), for the LC-graph (left) and C-graph (right) if the Real scenario occurs when considereing redistribution

**Fig. 14:**
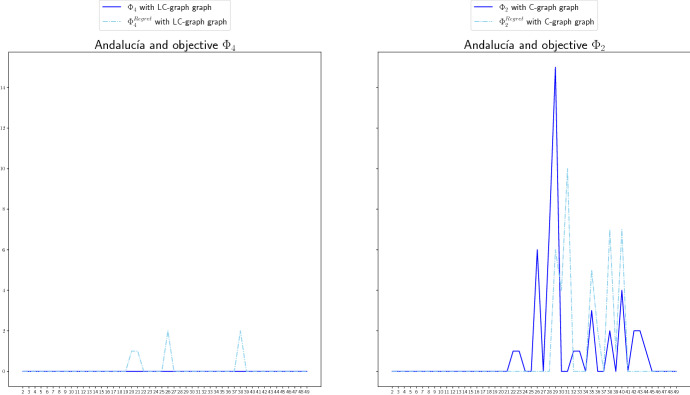
Non-covered demand in Andalucía, at each time period, for the regret (dashed line) and not regret (continuous line) versions of objective functions $\Phi _4$ (left) and $\Phi _2$ (right), for the LC-graph (right) and C-graph (left) if the Real scenario occurs when considereing redistribution

We compare in this last section the regret version (dashed line) versus the not regret version (continuous line) of different objective functions for Madrid in Fig. 13, and for Andalucía in Fig. 14. We can observe that for the cases of Madrid included in Fig. 14 (objective function $\Phi _2$ and LC-graph (left) and $\Phi _4$ and C-graph (right)) the total non-covered demand of each objective function practically coincides for the regret and not regret version. The reason for this could be that the existence of few available stock make that the redistribution options are scarce and similar in both cases. For the case of Andalucía, the version without regret tends to perform better for most of the cases if real scenario occurs. However, there exist cases, see for example the case of $\Phi _2$ vs $\Phi _2^\mathrm{Regret}$ and C-graph (right graphic), for which there exist periods that the regret version covers more demand than the version without regret.

In general, this tendency of the regret version to perform worse than the version without regret is maybe due to the election we made on the demand scenarios. Note that the real scenario, the one for which we are representing the NCD, is approximately an average of the other the other two scenarios, which benefits the objective functions without regret that average over the three scenarios. However, in cases in which the demand scenarios differ more, the regret version may perform better.

## Conclusions and further research

In this paper we propose decision aid tools to determine optimal distribution and sharing strategies in pandemic emergency situations to properly equip *expanded* hospitals and field hospitals. The approach is motivated by the COVID-19 pandemic in which, in its first wave, a lack of emergency health equipment provoked hospitals collapse, and many patients were not adequately treated. Our approach allows deciding on how to distribute equipment through a given network of units along a time horizon and how to share the extra stock received at some of the periods, such that a global measure of the (stochastic) demand of the units is minimized. We provide a unified Mixed Integer Linear Programming formulation for the problem, that allows to model different distribution networks and different objective (robust and min-max regret) functions. We also propose a divide-et-conquer math-heuristic approach for the problem that provides feasible solutions of the problem in reasonable computational times. Finally, we analyze the case of the lack of mechanical invasive ventilators during the COVID-19 health crisis in two different Spanish regions. We run our approach with different settings, obtaining as the main conclusion that applying our approach leads to a significant increase in the number of severe patients that can be rightly assisted. Furthermore, we observed that an optimal redistribution of the extra stock must be based on the demand and not on the distribution of population.

Some extensions of our approach will be the topic of a forthcoming paper. In particular, we observe that the use of logistic centers when distributing equipment in emergency situations may be advisable in many cases, since they allow a more adequate distribution, the non-used equipment storage, quicker deliveries, etc. However, some regions do not still have the infrastructures of those centers or the ones that they have are not sufficient. Also, in emergency situations, it may be useful to use *field logistic centers* during certain periods to improve the distribution of equipment during the demand peaks. In those cases, apart of deciding the amounts to be delivered and shared, one must decide where to locate new logistic centers. Our approach could be extended to this case with major modifications. In particular, the distribution network would not be known and is part of the decision (it depends on the position of the logistic centers), and then, it must be incorporated to the model, increasing considerably the complexity of the approach proposed in this paper. Observe that the model becomes a hub-and-spoke location problem in which the flows associated to commodities are decision variables of the model. Also, an interesting extension of this approach would be the incorporation of ordered weighted averaging aggregations of the non-covered demands to construct solutions under other robust objective functions (see e.g. Blanco et al. 2013, 2014, for an application of this type of aggregations in other logistic problems).

## Footnotes

1 Note that although overall non-covered expected demand is not formally a robust function, for the sake of generality we will also analyze this case.
